# Mapping the history and current situation of research on John Cunningham virus – a bibliometric analysis

**DOI:** 10.1186/1471-2334-9-28

**Published:** 2009-03-11

**Authors:** Hua-chuan Zheng, Lei Yan, Lei Cui, Yi-fu Guan, Yasuo Takano

**Affiliations:** 1 Department of Biochemistry and Molecular Biology, College of Basic Medicine, China Medical University, Shenyang, PR China; 2 Department of Medical Informatics and Information System, China Medical University, Shenyang, PR China; 3 Department of Diagnostic Pathology, Graduate School of Medicine and Pharmaceutical Sciences, University of Toyama, Toyama, Japan

## Abstract

**Background:**

John Cunningham virus (JCV) constitutes a family of polyoma viruses, which plays important roles in the progressive multifocal leukoencephalopathy (PML) and tumorigenesis. However, no bibliometric investigation has been reported to guide the researchers and potential readers.

**Methods:**

Papers were collected from database Sci-expanded and Pubmed until May 22, 2008. The highly-productive authors, institutes and countries, highly-cited authors and journals were ranked. The highly-cited articles were subjected to co-citation and chronological analysis with highly-frequent MeSH words for co-occurrence analysis.

**Results:**

Until now, 1785 articles about JCV were indexed in Sci-expanded and 1506 in Pubmed. The main document type was original article. USA, Japan and Italy were the largest three producers about JCV. Temple University published 128 papers and ranked the top, followed by University of Tokyo. Khalili K and Yogo Y became the core authors due to more than 20 documents produced. Journal of Neurovirology published more than 15 papers and ranked the top. Padgett BL and Berger JR were the first two highly-cited authors. Journal of Virology and Journal of Neurovirology respectively ranked to the first two highly-cited journals. These top highly-cited articles were divided into 5 aspects: (1) The correlation between JC virus and tumors; (2) Causal correlation of JCV with PML; (3) Polyoma virus infection and its related diseases in renal-allograft recipients; (4) Detection of JCV antibody, oncogene and its encoding protein; (5) Genetics and molecular biology of JCV. The MeSH/subheadings were classified into five groups: (1) JCV and virus infectious diseases; (2) JCV pathogenicity and pathological appearance of PML; (3) JCV isolation and detection; (4) Immunology of JCV and PML; (5) JCV genetics and tumors.

**Conclusion:**

JCV investigation mainly focused on its isolation and detection, as well as its correlation with PML and tumors. Establishment of transgenic animal model using JCV T antigen would be a hopeful and useful project in the further study.

## Background

John Cunningham virus (JCV) constitutes a family of polyoma viruses, which contain small, circular and double-stranded DNA genomes. The early region is alternatively spliced to produce large *T antigen *and small *t antigen *[1]. T antigen, a large nuclear phosphoprotein for viral DNA replication, binds to viral replication region to promote the unwinding of double helix and recruitment of cell proteins that are required for DNA synthesis. The late region encodes the capsid structural protein VP1, VP2 and VP3 due to alternative splicing and the small regulatory protein known as agnoprotein [1,2]. VP proteins are essential to assemble with viral DNA to form virons. Serological studies have indicated an asymptomatic JCV infection in about 90% of the adult population, but it may be activated under immunosuppressive conditions, leading to the lethal demyelinating disease, progressive multifocal leukoencephalopathy (PML) [1-5]. Evidences from transgenic and infectious animal models indicated that JCV could transform cells and cause various malignancies [6-9]. In recent years, links have been suggested between JCV and various types of human cancers, including colorectal, prostate and esophageal cancers, brain tumors, bronchopulmonary carcinoma and B cell lymphoma [1-9], pointing out its roles as oncovirus. However, no bibliometric investigation has been reported to guide the researchers and potential readers.

Investigators in some fields commonly predict that decision making for the following experiments, clinical practice and paper's submission should be based on the findings of scientific studies published in journals. Although scientific papers have provided useful and helpful information to the readers, it is a little difficult to learn about the history, status and future trend of some study field. The bibliometric method employs empiric data and quantitative analysis to trace the core production or citation, the content or quality of publications, and motivations of the researchers in the form of published literature so that it proves to be a valid and reliable way to map external and internal features of a scientific field [10]. A key assumption underpinning this method to catch insight into the flow of knowledge is that investigation papers represent knowledge produced by scientific research. Generally, academic productivity of individuals or groups is measured by counting the number of publications. The number of times that one work is cited is viewed as a measure of research impact. That is, the more frequently a paper is cited, the higher its impact or quality [10,11]. Examination of bibliometric information shows the communication patterns of the investigation within the field and the patterns of influence among different work. Authors who publish earlier and experience frequent citations tend to accrue the number of citations over time as Matthew effect describes. For example, co-citation analysis (in which two papers are cited together in a paper) can indicate a strong conceptual relationship between the studies. On the other hand, PubMed indexes journal articles using MeSH terms, which constitute a thesaurus that embodies all the concepts appearing in the medical literature and are arranged in a hierarchical, tree-like structure by subject categories. Associated with MeSH is a list of corresponding subheadings to enhance the focus of MeSH searches. The combination of MeSH terms and subheadings can not only facilitate the sensitivity and specificity of search, but also indicate the research contents and the relationship between papers [12-14]. If the further co-occurrence cluster analysis of MeSH is applied in some field, the close link between subtrees of the field will be well established.

In the present study, production and citation of JCV research have been analyzed using such bibliometric methods as chronological, co-citation and co-occurrence analysis to explore the whole history, current status and frontier about JCV study.

## Methods

### Data collection

The bibliographic data were collected in the database of the Institute for Scientific Information available on the web (http://www.isiknowledge.com, Sci-expanded) and National Library of Medicine on the web (http://www.ncbi.nlm.nih.gov/sites/entrez, Pubmed) until May 22, 2008. The tile, author, address, source, references or the US list of the papers were downloaded according to the retrieval strategy of "JC virus OR John Cunningham virus OR JCV OR JC polyomavirus OR JC polyoma virus" for Sci-expanded or Pubmed.

### Highly-produced and -cited analysis

Using Foxpro 5.0, Microsoft Excel, Bibliographic Item Co-occurrence Mining System (BIOCOMS) provided by Cui Lei and Sci-expanded statistical system, we applied Sci-expanded data to determine the document types, core authors, highly-produced institutes and countries. The references were analyzed to clarify the distribution of highly-cited papers, authors and journals. The top MeSH/subheading words were collected from Pubmed and subjected to statistical analysis for highly-frequent ones.

### Cluster analysis

After their identification, the top 34 most-cited articles were subjected to co-citation cluster analysis according to their co-citation times in one paper. The 48 highly-frequent MeSH/subheadings of all articles from Pubmed were studied using co-occurrence cluster analysis in term of their co-existence times in one paper. In any cluster analysis, the matrixes were built up according to co-citation or -occurrence times between the selected articles or words. Then, the related matrixes were developed using Ochiai index as previously described [15-18]. Finally, we employed the SPSS 10.0 software to perform the cluster analysis of these related matrixes.

## Results

### Core countries, institutes, authors and journals

**Figure 1:**
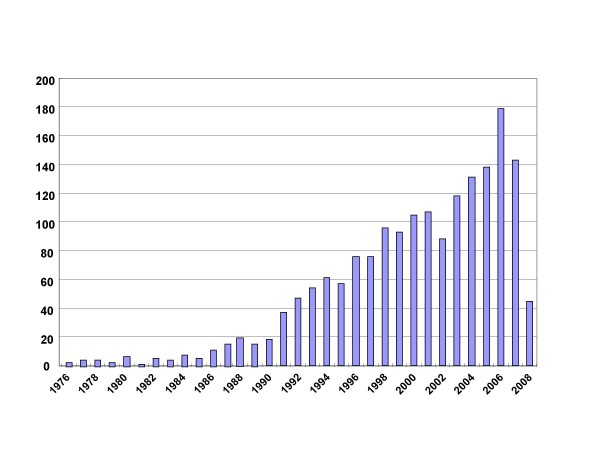
**Temporal distribution of production about JCV investigation**.

Until May 22, 2008, 1785 articles about JCV were indexed in Sci-expanded with 62508 references and 1506(225 reviews) in Pubmed with 6435 major MeSH/subheading words. The literature about JCV was gradually rising from 3 articles in 1976 until 179 in 2006 as indicated in Figure 1. The average annual growth rate was 5.7 pieces in the period. According to document type, there were 1307 original articles (73.2%), 123 reviews (6.9%) and 209 meeting abstracts (11.7%) in all collected literature (Table 1). In overall 21 countries listed, USA, Japan, Italy and Germany were in order the largest four producers about JCV despite 62 countries included (Table 2). The overall 1245 institutes were mentioned to investigate JCV, among which Temple University of USA published 128 papers and ranked the top, followed by University of Tokyo, and National Institute of Neurological Disorder and Stroke subsequently. Fourteen of 21 (66.7%) core institutes come from USA with three core institutes in Italy (Table 3). Such 33 authors as Khalili K, Yogo Y and etc produced more than 20 documents in spite of all 4856 authors involved. There were 9 highly-produced scientists from Temple University and 6 from University of Tokyo, Japan, and 4 from National Institute of Neurological Disorder and Stroke, USA respectively (Table 4). As shown in Table 5, Journal of Neurovirology, Journal of Virology, Virology, Journal of Medical Virology, Journal of General Virology and so forth published more than 15 papers and were considered as the core journals although there existed JCV papers in 395 journals. These source journals mainly include the field of Virology, Neurosciences, Clinical Neurology, Immunology, Pathology, Oncology and so on (Table 6).

**Table 1:** Document types of the scientific papers about JCV

Num	Document type	Record count	Percentage(%)
1	Original article	1307	73.2
2	Meeting abstract	209	11.7
3	Review	123	6.9
4	Note	63	3.5
5	Letter	40	2.2
6	Editorial material	31	1.7
7	Correction	4	0.20
8	Book review	3	0.17
9	Discussion	2	0.11

**Table 2:** The territory distribution of the scientific papers about JCV

Num	Country	Record count	Percentage(%)
1	USA	968	54.2
2	Japan	190	10.6
3	Italy	173	9.7
4	Germany	158	8.8
5	UK	88	5.0
6	France	72	4.0
7	Spain	48	2.7
8	Switzerland	44	2.5
9	Canada	37	2.1
10	Sweden	36	2.0
11	Taiwan	24	1.3
12	Norway	22	1.2
13	China	15	0.8
14	Australia	14	0.8
15	Poland	13	0.7
16	Belgium	12	0.7
17	Brazil	12	0.7
18	South Korea	12	0.7
19	Lithuania	11	0.6
20	Finland	10	0.6
21	Netherlands	10	0.6

**Table 3:** The core institutes to investigate the JCV

Num	Institution name	Country	Record count	Percentage(%)
1	Temple University	USA	128	7.2
2	University of Tokyo	Japan	86	4.8
3	National Institute of Neurological and Communication Disorders and Stroke	USA	79	4.4
4	Harvard University	USA	61	3.4
5	Hokkaido University	Japan	59	3.3
6	National Institute of Neurological Disorder and Stroke	USA	56	3.1
7	Pennsylvania State University	USA	56	3.1
8	Thomas Jefferson University	USA	47	2.6
9	University of Milan	Italy	40	2.2
10	University of Wisconsin	USA	39	2.2
11	Brown University	USA	38	2.1
12	Johns Hopkins University	USA	36	2.0
13	National Cancer Institute	USA	32	1.8
14	University of California san Diego	USA	31	1.7
15	University of Wurzburg	Germany	29	1.6
16	University of Pittsburgh	USA	28	1.6
17	IRCCS	Italy	25	1.4
18	Baylor College of Medicine	USA	24	1.3
19	University of Hamburg	Germany	24	1.3
20	National Institute of Health	USA	23	1.3
21	University of Ferrara	Italy	20	1.1

**Table 4:** The core authors for JCV investigation

Num	Core author	Institutes	Record count	Percentage(%)
1	Khalili K	Temple University, USA	174	9.8
2	Yogo Y	University of Tokyo, Japan	75	4.2
3	Major EO	National Institute of Neurological Disorders and Stroke, USA	74	4.2
4	Stoner GL	National Institute of Neurological Disorders and Stroke, USA	70	3.9
5	Del Valle L	Temple University, USA	62	3.5
6	Kitamura T	University of Tokyo, Japan	59	3.3
7	Nagashima K	Hokkaido University, Japan	57	3.2
8	Frisque RJ	Pennsylvania State University, USA	48	2.7
9	Ryschkewitsch CF	National Institute of Neurological Disorders and Stroke, USA	45	2.5
10	Koralnik IJ	Harvard Medical School, USA	43	2.4
11	Gordon J	Temple University, USA	42	2.4
12	Sugimoto C	University of Tokyo, Japan	41	2.3
13	Atwood WJ	Brown University, USA	40	2.2
14	Walker DL	University of Wisconsin Medical School, USA	39	2.2
15	Zheng HY	University of Tokyo, Japan	37	2.1
16	Ferrante P	University of Milan, Italy	33	1.9
17	Sawa H	Hokkaido University, Japan	32	1.8
18	Agostini HT	National Institute of Neurological Disorders and Stroke, USA	28	1.6
19	Boland CR	Baylor University Medical Center, USA	27	1.5
20	Takasaka T	University of Tokyo, Japan	26	1.5
21	White MK	Temple University, USA	26	1.5
22	Croul S	Temple University, USA	25	1.4
23	Safak M	Temple University, USA	25	1.4
24	Wegner M	Universität Hamburg, Germany	25	1.4
25	Amini S	Temple University, USA	24	1.3
26	Padgett BL	University of Wisconsin Medical School, USA	23	1.3
27	Reiss K	Temple University, USA	22	1.2
28	Tanaka S	Hokkaido University, Japan	22	1.2
29	Berger JR	Temple University, USA	21	1.2
30	Butel JS	Baylor University Medical Center, USA	21	1.2
31	Dorries K	Universität Würzburg, Germany	21	1.2
32	Shah KV	Johns Hopkins Bloomberg School of Public Health, USA	21	1.2
33	Ikegaya H	University of Tokyo, Japan	20	1.1

**Table 5:** Core journals of JCV investigation

Num	Source title	Record count	Percentage(%)
1	Journal of Neurovirology	167	9.4
2	Journal of Virology	128	7.2
3	Virology	69	3.9
4	Journal of Medical Virology	51	2.9
5	Journal of General Virology	44	2.5
6	Annals of Neurology	36	2.0
7	Journal of Infectious Diseases	35	2.0
8	Journal of Clinical Micrology	31	1.7
9	Journal of Neuropathology and Experimental Neurology	31	1.7
10	AIDS	29	1.6
11	Journal of Biological Chemistry	29	1.6
12	Neurology	29	1.6
13	Oncogene	25	1.4
14	Proceedings of The National Academy of Sciences of The United States of America	25	1.4
15	Journal of Virological Methods	24	1.3
16	Transplantation	23	1.3
17	Gastroenterology	21	1.2
18	Anthropological Science	20	1.1
19	International Journal of Cancer	20	1.1
20	Polyomaviruses and Human Diseases	19	1.1
21	Archives of Virology	17	1.0
22	Clinical Infectious Diseases	16	0.9
23	American Journal of Transplantation	15	0.8
24	New England Journal of Medicine	15	0.8

**Table 6:** Subject categories for JCV investigation

Num	Subject category	Record count	Percentage (%)
1	Virology	601	33.7
2	Neurosciences	329	18.4
3	Clinical Neurology	238	13.3
4	Immunology	154	8.6
5	Pathology	154	8.6
6	Oncology	144	8.1
7	Biochemistry & Molecular Biology	142	8.0
8	Infectious Diseases	128	7.2
9	Biotechnology & Applied Microbiology	103	5.8
10	Cell Biology	85	4.8
11	Microbiology	80	4.5
12	Transplantation	80	4.5
13	Surgery	73	4.1
14	Genetics & Heredity	61	3.4
15	Medicine, Research & Experimental	60	3.4
16	Medicine, General & Internal	58	3.3
17	Evolutionary Biology	40	2.2
18	Hematology	35	2.0
19	Urology & Nephrology	34	1.9
20	Pediatrics	33	1.9
21	Biochemical Research Methods	32	1.8
22	Gastroenterology & Hepatology	31	1.7
23	Multidisciplinary Sciences	31	1.7

### Highly-cited authors, journals and papers

The papers of 10 highly-cited authors (totally 1577 producers) like Padgett BL and Berger JR were cited for more than 400 times, among whom 8 persons come from USA (Table 7). The 10 highly-cited journals (totally 3584 journals) were selected due to more than 1179 citation times, including 3 for Virology and 4 for comprehensive journals (Table 8). Journal of Virology and Journal of Neurovirology respectively ranked to the first two among 404 cited journals (Table 9). The highly-cited papers were chronologically analyzed and grouped into two stages: (1) 1971–1984: discovery and isolation of JCV in PML disease and (2) 1985-present: clarification of JCV genomic DNA sequence and its correlation with diseases (Table 9).

**Table 7:** The highly-cited authors for JCV papers

Num	Authors	Institute	CT	CP(%)
1	Padgett BL	University of Wisconsin Medical School, USA	958	1.53
2	Berger JR	University of Kentucky, USA	761	2.75
3	Agostini HT	National Institute of Neurological Disorders and Stroke, USA	649	3.79
4	Frisque RJ	Pennsylvania State University, USA	630	4.80
5	Major EO	National Institute of Neurological Disorders and Stroke, USA	604	5.77
6	Walker DL	Tulane University, USA	495	6.56
7	Arthur RR	Johns Hopkins University, USA	430	7.25
8	Dorries K	University of Wurzburg, Germany	419	7.92
9	Yogo Y	University of Tokyo, Japan	415	8.58
10	Shah KV	Johns Hopkins University, USA	400	9.23

CT, cited time; CP, cumulative percentage

**Table 8:** The highly-cited journals for JCV papers

Num	Journal	CT	CP(%)
1	Journal of Virology	5871	9.43
2	Virology	2498	13.44
3	Proceedings of The National Academy of Sciences of The United States of America	2313	17.16
4	Journal of Infectious Diseases	2230	20.74
5	New England Journal of Medicine	1655	23.40
6	Journal of Neurovirology	1274	25.45
7	Science	1230	27.42
8	Cell	1227	29.39
9	Journal of Clinical Microbiology	1201	31.32
10	Annals of Neurology	1179	33.22

CT, cited time; CP, cumulative percentage

**Table 9:** The highly-cited articles for the JCV investigation

N	Authors	Year	Title	Source	V	P	CT	CP
1	Padgett BL	1971	Cultivation of papova-like virus from human brain with progressive multifocal leucoencephalopathy.	Lancet	1	1257	366	0.59
2	Frisque RJ	1984	Human polyomavirus JC virus genome.	J Virol	51	458	360	1.16
3	Major EO	1992	Pathogenesis and molecular biology of progressive multifocal leukoencephalopathy, the JC virus-induced demyelinating disease of the human brain	Clin Microbiol Rev	5	49	285	1.62
4	Padgett BL	1973	Prevalence of antibodies in human sera against JC virus, an isolate from a case of progressive multifocal leukoencephalopathy	J Infect Dis	127	467	219	1.97
5	Chesters PM	1983	Persistence of DNA sequences of BK virus and JC virus in normal human tissues and in diseased tissues.	J Infect Dis	147	676	215	2.31
6	Berger JR	1987	Progressive multifocal leukoencephalopathy associated with human immunodeficiency virus infection. A review of the literature with a report of sixteen cases.	Ann Intern Med	107	78	197	2.63
7	Astrom KE	1958	Progressive multifocal leuko-encephalopathy; a hitherto unrecognized complication of chronic lymphatic leukaemia and Hodgkin's disease	Brain	81	93	179	2.91
8	Yogo Y	1990	Isolation of a possible archetypal JC virus DNA sequence from nonimmunocompromised individuals.	J Virol	64	3139	172	3.19
9	Gardner SD	1971	New human papovavirus (B.K.) isolated from urine after renal transplantation	Lancet	1	1253	165	3.45
10	Tornatore C	1992	Detection of JC virus DNA in peripheral lymphocytes from patients with and without progressive multifocal leukoencephalopathy	Ann Neurol	31	454	146	3.69
11	Kitamura T	1990	High incidence of urinary JC virus excretion in nonimmunosuppressed older patients.	J Infect Dis	161	1128	136	3.90
12	Houff SA	1988	Involvement of JC virus-infected mononuclear cells from the bone marrow and spleen in the pathogenesis of progressive multifocal leukoencephalopathy.	New Engl J Med	318	301	135	4.12
13	Berger JR	1995	Progressive multifocal leukoencephalopathy: the evolution of a disease once considered rare	J Neurovirol	1	5	134	4.33
14	Arthur RR	1989	Detection of BK virus and JC virus in urine and brain tissue by the polymerase chain reaction.	J Clin Microbiol	27	1174	132	4.55
15	Walker DL	1973	Human papovavirus (JC): induction of brain tumors in hamsters	Science	181	674	128	4.75
16	Loeber G	1988	DNA rearrangements in organ-specific variants of polyomavirus JC strain GS	J Virol	62	1730	126	4.95
17	White FA	1992	JC virus DNA is present in many human brain samples from patients without progressive multifocal leukoencephalopathy	J Virol	66	5726	117	5.14
18	Agostini HT	1996	Genotype profile of human polyomavirus JC excreted in urine of immunocompetent individuals.	J Clin Microbiol	34	159	114	5.32
19	Bergsagel DJ	1992	DNA sequences similar to those of simian virus 40 in ependymomas and choroid plexus tumors of childhood	New Engl J Med	326	988	108	5.49
20	Brooks BR	1984	Progressive multifocal leukoencephalopathy	Neurol Clin	2	299	108	5.67
21	Markowitz RB	1993	Incidence of BK virus and JC virus viruria in human immunodeficiency virus-infected and -uninfected subjects.	J Infect Dis	167	13	108	5.84
22	Kenney S	1984	Prospective study of the human polyomaviruses BK and JC and cytomegalovirus in renal transplant recipients.	Science	226	1337	107	6.01
23	London WT	1978	Brain tumors in owl monkeys inoculated with a human polyomavirus (JC virus).	Science	201	1246	106	6.18
24	Coleman DV	1980	A prospective study of human polyomavirus infection in pregnancy	J Infect Dis	142	1	105	6.35
25	Dorries K	1994	Infection of human polyomaviruses JC and BK in peripheral blood leukocytes from immunocompetent individuals.	Virology	198	59	105	6.52
26	Gardner SD	1984	Prospective study of polyomavirus type BK replication and nephropathy in renal-transplant recipients	J Clin Pathol	37	578	104	6.68
27	Flaegstad T	1991	Amplification and sequencing of the control regions of BK and JC virus from human urine by polymerase chain reaction	Virology	180	553	102	6.85
28	Hogan TF	1980	Human polyomavirus infections with JC virus and BK virus in renal transplant patients	Ann Intern Med	92	373	101	7.01
29	Martin JD	1985	Differences in regulatory sequences of naturally occurring JC virus variants	J Virol	53	306	97	7.16
30	Monaco MCG	1996	JC virus infection of hematopoietic progenitor cells, primary B lymphocytes, and tonsillar stromal cells: implications for viral latency.	J Virol	70	7004	96	7.32
31	Richardson EP	1961	Progressive multifocal leukoencephalopathy	New Engl J Med	265	815	95	7.47
32	Randhawa PS	1999	Human polyoma virus-associated interstitial nephritis in the allograft kidney.	Transplantation	67	103	92	7.62
33	Frisque RJ	1992	The molecular biology of JC virus, causative agent of progressive multifocal leukoenchephalpathy	Mol Neurovirology		25	92	7.76
34	Padgett BL	1977	JC virus, a human polyomavirus associated with progressive multifocal leukoencephalopathy: additional biological characteristics and antigenic relationships	Infect Immun	15	656	90	7.91

V, volume; P, page; CT, cited times; CP, cumulative percentage

### Co-citation analysis of highly-cited articles

**Figure 2:**
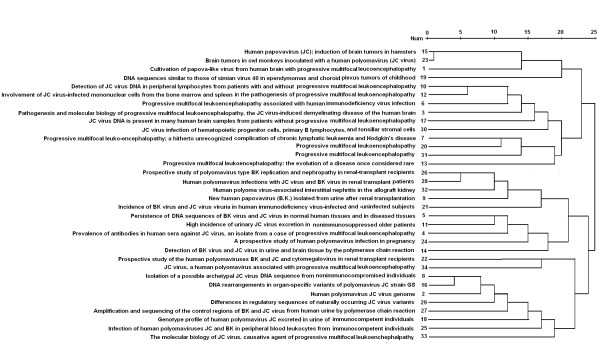
**Co-citation cluster analysis of highly-cited references**.

In the overall references about JCV, most highly-cited articles were published before 1999 with more than 90 citation times and came from major journals, such as Journal of Infectious Disease, Journal of Virology, Science, New England Journal of Medicine and so forth. As shown in Figure 2, these top highly-cited articles were divided into 5 aspects by co-citation analysis: (1) The correlation between JC virus and tumors; (2) Causal correlation of JCV with PML: pathogenesis and molecular biology; (3) Polyoma virus infection and its related diseases in renal-allograft recipients; (4) Detection of JCV antibody, gene and encoding protein; (5) Genetics and molecular biology of JCV.

### Co-occurrence analysis of highly-frequent MeSH/subheading words

**Figure 3:**
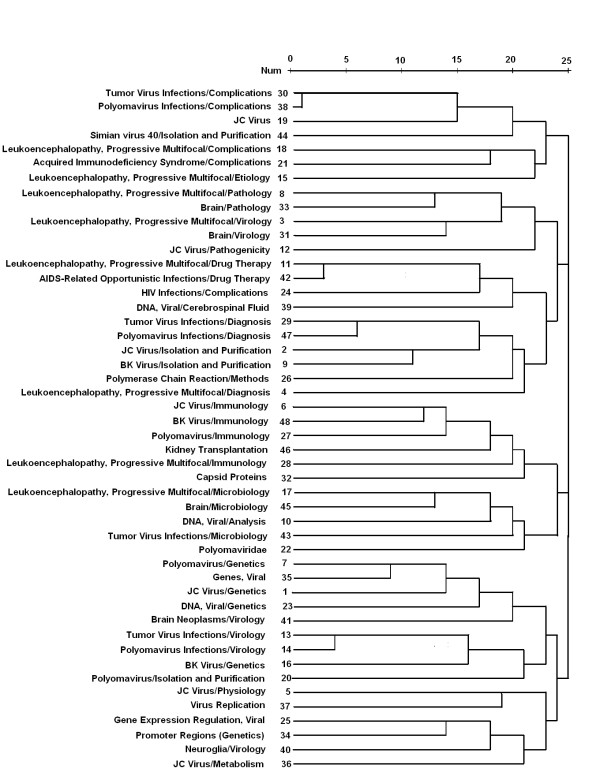
**Co-occurrence cluster analysis of the highly-frequent MeSH/subheading words**.

The 48 highly-frequent MeSH/subheading words generally existed for more than 25 times in the papers about JCV (Table 10). Among them, 17 words (35.4%) belonged to the C02 subcategory of MeSH (Viral Disease) and 15 (31.3%) to B04 subcategory (Viruses). These MeSH/subheadings were classified into five groups: (1) JCV and virus infectious diseases; (2) JCV pathogenicity and pathological appearance of PML; (3) JCV isolation and detection; (4) Immunology of JCV and PML; (5) JCV genetics and tumors(Figure 3).

**Table 10:** The highly-frequent MeSH/subheading words

Num	MeSH/subheading words	Subcategory number	Times	CP
1	JC Virus/Genetics	B04.280.640.615.400	289	4.49
2	JC Virus/Isolation and Purification	B04.280.640.615.400	216	7.84
3	Leukoencephalopathy, Progressive Multifocal/Virology	C02.182.500.300.500	115	9.63
4	Leukoencephalopathy, Progressive Multifocal/Diagnosis	C02.182.500.300.500	86	10.96
5	JC Virus/Physiology	B04.280.640.615.400	84	12.27
6	JC Virus/Immunology	B04.280.640.615.400	73	13.40
7	Polyomavirus/Genetics	B04.280.640.615	69	14.47
8	Leukoencephalopathy, Progressive Multifocal/Pathology	C02.182.500.300.500	68	15.53
9	BK Virus/Isolation and Purification	B04.280.640.615.100	68	16.58
10	DNA, Viral/Analysis	D13.444.308.568	64	17.57
11	Leukoencephalopathy, Progressive Multifocal/Drug Therapy	C02.182.500.300.500	58	18.48
12	JC Virus/Pathogenicity	B04.280.640.615.400	56	19.34
13	Tumor Virus Infections/Virology	C02.928	54	20.18
14	Polyomavirus Infections/Virology	C02.256.721	51	20.98
15	Leukoencephalopathy, Progressive Multifocal/Etiology	C02.182.500.300.500	50	21.75
16	BK Virus/Genetics	B04.280.640.615.100	50	22.53
17	Leukoencephalopathy, Progressive Multifocal/Microbiology	C02.182.500.300.500	47	23.26
18	Leukoencephalopathy, Progressive Multifocal/Complications	C02.182.500.300.500	45	23.96
19	JC Virus	B04.280.640.615.400	45	24.65
20	Polyomavirus/Isolation and Purification	B04.280.640.615	42	25.31
21	Acquired Immunodeficiency Syndrome/Complications	C02.782.815.616.400.040	39	25.91
22	Polyomaviridae	B04.280.640	38	26.50
23	DNA, Viral/Genetics	D13.444.308.568	37	27.08
24	HIV Infections/Complications	C02.782.815.616.400	36	27.64
25	Gene Expression Regulation, Viral	G05.315.385	35	28.18
26	Polymerase Chain Reaction/Methods	E05.393.620.500	34	28.71
27	Polyomavirus/Immunology	B04.280.640.615	34	29.23
28	Leukoencephalopathy, Progressive Multifocal/Immunology	C02.182.500.300.500	34	29.76
29	Tumor Virus Infections/Diagnosis	C02.928	33	30.27
30	Tumor Virus Infections/Complications	C02.928	33	30.79
31	Brain/Virology	A08.186.211	33	31.30
32	Capsid Proteins	D12.776.964.970.600.550	33	31.81
33	Brain/Pathology	A08.186.211	31	32.29
34	Promoter Regions (Genetics)	G06.184.603.080.689.675	31	32.77
35	Genes, Viral	G14.330.605	30	33.24
36	JC Virus/Metabolism	B04.280.640.615.400	30	33.71
37	Virus Replication	G04.185.515.880.941	29	34.16
38	Polyomavirus Infections/Complications	C02.256.721	28	34.59
39	DNA, Viral/Cerebrospinal Fluid	D13.444.308.568	28	35.03
40	Neuroglia/Virology	A08.637	28	35.46
41	Brain Neoplasms/Virology	C04.588.614.250.195	27	35.88
42	AIDS-Related Opportunistic Infections/Drug Therapy	C01.539.597.050	27	36.30
43	Tumor Virus Infections/Microbiology	C02.928	27	36.72
44	Simian virus 40/Isolation and Purification	B04.280.640.615.700	27	37.14
45	Brain/Microbiology	A08.186.211	25	37.53
46	Kidney Transplantation	E02.870.500	25	37.91
47	Polyomavirus Infections/Diagnosis	C02.256.721	25	38.30
48	BK Virus/Immunology	B04.280.640.615.100	25	38.69

CP, cumulative percentage

## Discussion

A systematic view of JCV papers to discern the distinct set of core researchers, institutional affiliations and corresponding countries helps us to gain a deeper understanding of approaches to JCV. As shown in our bibliometric analysis, the document type of JCV was original articles (1307/1785) and many data (209/1785) had been communicated in meeting activities. The review part occupies 6.9% (123/1785). The results indicated that JCV research was very active and interesting many investigators, and some scientists had begun to summary the achievement of JCV. Among 33 core authors, 19 persons come from Temple University, University of Tokyo, and National Institute of Neurological Disorder and Stroke, which ranked the top in the highly-produced institutes. Additionally, 14 (66.7%) core institutes of USA also focused on the investigation of JCV and USA was the first top producer of JCV papers until now. JVC was discovered in 1971 by American Padgett and named after the two initials of a patient with progressive multifocal leukoencephalopathy (PML). It was suggested that the JCV investigation originated from USA, which consequently became the top source information for JCV. It is rational and helpful for the scientists to tack the core authors and institutes to grasp the frontier of this field, open new projects and submit their distinguished work.

The list of top-cited articles about JCV identified the authors, articles and topics that reflected history and development of this specialty. Among highly-cited authors, Padgett is the discoverer for JCV in PML and published the first article in Lancet. The paper has been cited for 366 times and ranks the top in the highly-cited ones. His outstanding was also due to another article in Journal of Infectious disease, which described the detection of the antibody against JCV in PML. Therefore, it is explanatory for Padgett BL to be the most highly cited. These top-cited articles produced valuable information for readers, but also tell us some historical achievement in some field. According to these highly-cited papers, the research about JCV was chronologically separated into beginning and developing stages including discovery and isolation of JCV in PML disease, and clarification of JCV genomic DNA sequence and its relationship with diseases by polymerase chain reaction (PCR) respectively.

Most of highly-cited journals almost come from Virology, Neurology, and comprehensive journals, indicating JCV paper mainly absorbs frontier knowledge from these fields. Oncogene, Journal of Biological Chemistry, and International Journal of Cancer also become the highly-cited journal (data not shown), indicating the attempts of JCV study to combine with Molecular Biology and Oncology. This data also demonstrate the close link of JCV with these specialties. In the overall references of JCV papers, most highly-cited articles were published in Proceeding of National Academy and Science, USA and New England Journal of Medicine, indicating that these famous-brand journals highlight the investigation of JCV and emphasized the scientific achievement of JCV. Therefore, investigators of JCV not only read the journals of Virology, but also emphasized the novel findings of JCV published in other journals with high impact factor.

Methodologically, the cluster techniques include text segmentation, summary extraction, feature selection, term association, cluster generation, topic identification, and information mapping [19]. Clustering algorithms prominently used in co-citation analysis has proved very useful in revealing research streams in some discipline [20-23]. Here, we carried out empirical co-citation analysis to map the network of highly-cited papers about JCV. Our data indicated that these top highly-cited articles were grouped into such 4 aspects as the correlation between JC virus and tumors, causal correlation of JCV with PML, polyoma virus infection and its related diseases in renal-allograft recipients, detection of JCV antibody, oncogene and its encoding protein, and genetics and molecular biology of JCV. These findings might not only enrich the knowledge of students and specialists about the development's history of JCV research, but also open new bursts of scientific investigation.

Co-occurrence has been considered as carriers of meaning across different domains in studies of science. Based on this principle, we performed co-occurrence cluster analysis using Pubmed MeSH/subheading words to construct a new tie between two words depending on the co-existing frequencies [24]. Consequently, most of the top highly-frequent MeSH/subheading words are mainly classified into C02 subcategory of MeSH (Viral Disease) and B04 subcategory (Viruses). The analytic data showed that the contents of published papers about JCV included JCV isolation and detection, as well as JCV and virus infectious diseases like PML or tumors. It was suggested that JCV investigation centered on its isolation, its pathogenicity of PML and its genetics at early time. Recently, the causal relationship between JCV and tumors has been emphasized by the scientists. It was demonstrated that JCV investigation like isolation and detection mainly aimed to clarify the molecular mechanism of its relevant diseases including PML and tumors.

As well known, JCV infection experiences two outcomes as other viruses. In un-permissive condition, JCV infection initiates binding to the JCV-sensitive cell surface and JCV capsids undergo endocytosis and are transported to the nucleus where the viral DNA is uncoated and the early and late region begins to be transcripted. Subsequently, JCV genomic DNA is assembled with caspid protein to undergo the lytic viral release, finally to cause demyelinating disease, PML. Under permissive infection, viral DNA can replicate, resulting in lytic infection with viral amplification and non-permissive cells don't allow the viral replication, leading to an abortive infection or cell transformation [6-9]. The evidence provided enough reasons for the following data: (1) The core and highly-cited journals mainly contained the field of virology, neurology and oncology; (2) The highly-cited articles and highly-frequent MeSH/subheading also mentioned the research contents of JCV, PML and tumors.

Recently, the further clarification of JCV genetics promoted the scientists to detect its genomic existence in tumors or make the transgenic mice to study the oncogenic role of JCV. Our group had examined the JCV targeting T antigen using nested-PCR, real-time PCR, in situ PCR, in situ hybridization, and immunohistochemistry [6-9]. It was found that positive rate and copies of JCV were higher in gastric, lung and tongue carcinomas than corresponding normal tissues, indicating its oncogenic role in epithelial carcinogenesis. Furthermore, JCV T antigen can serve as helicase, and polymerase, orchestrate the assembly and function of cellular proteins, disrupt the signal pathways of p53, Rb and Wnt signaling pathway, and should be considered as a viral oncogene [2-4]. Therefore, we are establishing a transgenic model of gastric neoplasia induced by JCV T antigen, which will help to verify the oncogenic role of JCV in gastric carcinoma and provide a novel tool to investigate gastric carcinomas. It was hypothesized that application of JCV T antigen in tumor transgenic animal model would be a novel and hot project in the future.

## Conclusion

In this study, we successfully performed the scientometric analysis of JCV literature. Our data indicated that JCV mainly centered on PML and tumors. The bibliometric study assists researchers to know the history and frontier of JCV investigation, guide them to open new projects and submit the distinguished work. These cluster methods employed in this investigation can clarify the history, status and development in the field of JCV.

## Competing interests

The authors declare that they have no competing interests.

## Authors' contributions

HCZ conceived the study and wrote the first and final draft of the manuscript, analyzed interpretation. LY extracted the data and performed the analyses. LC kindly provided the bibliometric software and kindly guidance. YFG and YT gave many good suggestions about data processing and manuscript. All authors read and approved the final manuscript.

## Pre-publication history

The pre-publication history for this paper can be accessed here:

http://www.biomedcentral.com/1471-2334/9/28/prepub
